# Effects of Ca Content on Formation and Photoluminescence Properties of CaAlSiN_3_:Eu^2+^ Phosphor by Combustion Synthesis

**DOI:** 10.3390/ma9030178

**Published:** 2016-03-08

**Authors:** Shyan-Lung Chung, Shu-Chi Huang

**Affiliations:** 1 Department of Chemical Engineering, National Cheng Kung University, Tainan 70101, Taiwan; n38981266@mail.ncku.edu.tw; 2 Advanced Optoelectronic Technology Center, National Cheng Kung University, Tainan 70101, Taiwan

**Keywords:** nitride phosphor, CaAlSiN_3_:Eu^2+^, combustion synthesis, white light LED

## Abstract

Effects of Ca content (in the reactant mixture) on the formation and the photoluminescence properties of CaAlSiN_3_:Eu^2+^ phosphor (CASIN) were investigated by a combustion synthesis method. Ca, Al, Si, Eu_2_O_3_, NaN_3_, NH_4_Cl and Si_3_N_4_ powders were used as the starting materials and they were mixed and pressed into a compact which was then wrapped up with an igniting agent (*i.e*., Mg + Fe_3_O_4_). The compact was ignited by electrical heating under a N_2_ pressure of ≤1.0 MPa. By keeping the molar ratios of Al and Si (including the Si powder and the Si in Si_3_N_4_ powder) both at 1.00 and that of Eu_2_O_3_ at 0.02, XRD (X-ray diffraction) coupled with TEM-EDS (transmission electron microscope equipped with an energy-dispersive X-ray spectroscope) and SAED (selected area electron diffraction) measurements show that AlN:Eu^2+^ and Ca-α-SiAlON:Eu^2+^ are formed as the major phosphor products when the Ca molar ratio (denoted by *Y*) is equal to 0.25 and AlN:Eu^2+^ and Ca-α-SiAlON:Eu^2+^ could not be detected at *Y* ≥ 0.75 and ≥1.00, respectively. CASIN (*i.e*., CaAlSiN_3_:Eu^2+^) becomes the only phosphor product as *Y* is increased to 1.00 and higher. The extent of formation of CASIN increases with increasing *Y* up to 1.50 and begins to decrease as *Y* is further increased to 1.68. While the excitation wavelength regions are similar at various *Y*, the emission wavelength regions vary significantly as *Y* is increased from 0.25 to 1.00 due to different combinations of phosphor phases formed at different *Y*. The emission intensity of CASIN was found to vary with *Y* in a similar trend to its extent of formation. The Ca and Eu contents (expressed as molar ratios) in the synthesized products were found to increase roughly with increasing *Y* but were both lower than the respective Ca and Eu contents in the reactant mixtures.

## 1. Introduction

White light LED lighting is expected to become the major lighting technique in the next generation due to its advantages such as energy efficiency, long lifetime, compactness, environment friendliness and designable features [1,2]. Currently, white LED lighting devices are mostly fabricated based on the combination of an InGaN-based blue-LED chip and a yellow-emitting phosphor, *i.e.*, YAG:Ce^3+^[3,4]. Among several problems which have been found with this type of LED lighting devices, the ones related directly to the phosphor are mainly the need for improvement of thermal stability, quantum efficiency and color rendering index (due to deficiency of red light) [4].

In the past decade, a new class of phosphors (*i.e*., rare-earth doped nitridosilicates) has been discovered and shown to be ideal for application in LED lighting due to their superior properties such as high quantum efficiency, red light emission and high thermal and chemical stability [5]. Two major types of nitridosilicate have been developed as the host lattices for the nitridosilicate phosphors, namely alkaline-earth silicon nitrides (M_2_Si_5_N_8_, M = Ca, Sr and Ba, often referred to as 2-5-8 phosphors) and alkaline-earth aluminum silicon nitrides (MAlSiN_3_, M = Mg, Ca and Sr, often referred to as 1-1-1-3 phosphors). Many methods have been developed for the synthesis of nitridosilicate phosphors including solid state reaction (SSR) [6], carbothermal reduction and nitridation (CRN) [7], gas pressure sintering [8], and combustion synthesis (SHS) [9]. However, many of these methods utilize costly and oxygen or moisture sensitive chemicals as the starting materials, and most of the methods are carried out under severe synthesis conditions (e.g., high temperatures, high pressures and long reaction time).

In our previous study [10], a combustion synthesis method was developed for the synthesis of Eu^2+^-doped CaAlSiN_3_ phosphor: Ca, Al, Si and Eu_2_O_3_ powders were used as the Ca, Al, Si and Eu sources and NaN_3_, NH_4_Cl and Si_3_N_4_ were added to enhance the product yield. The synthesis reaction was triggered by the combustion of an igniting agent wrapping up the reactant compact. A product yield of ~71% was obtained under a N_2_ pressure of 0.7 MPa. In addition to easy handling of the reactants and a low N_2_ pressure required (~0.7 MPa), the method developed possesses many other advantages including simple and inexpensive equipment required, relatively low cost of the reactants, fast reaction and short processing time, potential capability for mass production and possibly low production costs.

It is known that Eu^2+^ doped CaAlSiN_3_ phosphor (simply referred to as CASIN hereafter), Eu^2+^ is incorporated into the host lattice at the Ca^2+^ sites (*i.e*., by substituting for Ca^2+^) [1,2]. Suehiro *et al*. [11] reported that the molar ratios of the metals (*i.e*., Ca) in their synthesized CASIN were all below the stoichiometric values. An interesting problem thus arises that how the metal molar ratios affect the photoluminescence properties, crystallinity and morphology of CASIN. However, this problem has been rarely studied [11,12] especially for the effects of the molar ratio of Ca^2+^.

Our research has been aimed at studying the effects of the molar ratio of Ca^2+^ on the photoluminescence properties of CASIN by employing the combustion synthesis method developed in our previous study [10] for the synthesis of CASIN. Since the molar ratio of Ca^2+^ in CASIN is very difficult to be controlled and measured precisely, it is simply adjusted by varying the content of Ca powder in the reactant mixture. In this work, we report an experimental study on the effects of Ca content (in the reactant mixture) on the formation and the photoluminescence properties of CASIN.

## 2. Results and Discussion

### 2.1. Effects of Ca Content on Product Formation and Morphology

**Figure 1:**
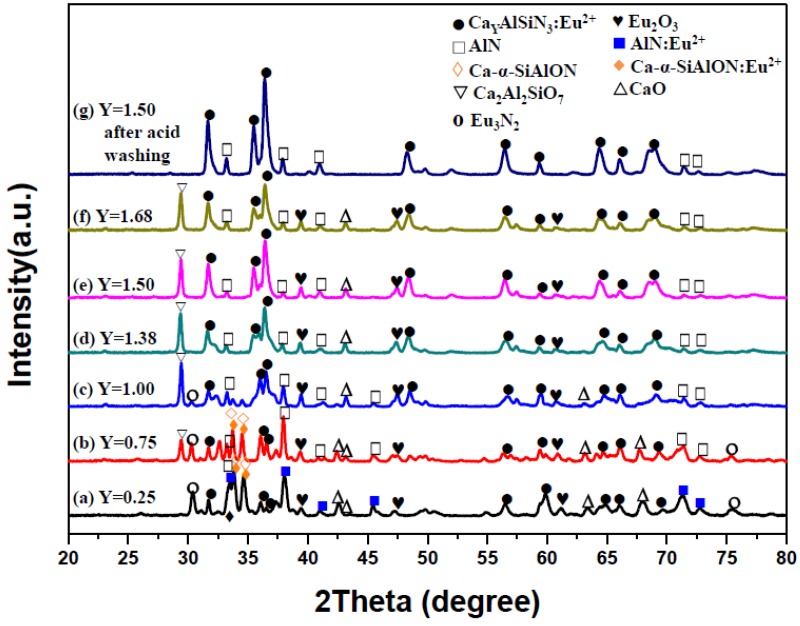
XRD patterns of the as-synthesized products obtained with *Y* = 0.25 (**a**); 0.75 (**b**); 1.00 (**c**); 1.38 (**d**); 1.50 (**e**); and 1.68 (**f**); and a product obtained with *Y* = 1.50 after acid washing (**g**).

**Figure 2:**
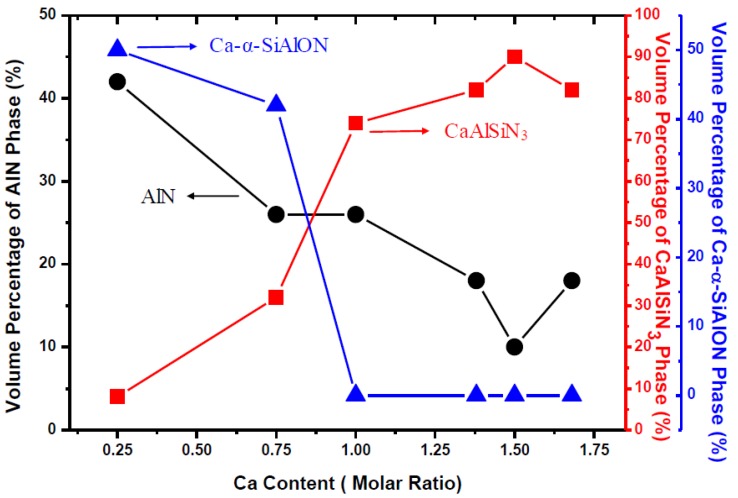
Volume Percentages of AlN, CaAlSiN_3_ and Ca-α-SiAlON phases formed during synthesis with various Ca contents.

Figure 1a–f are the XRD patterns of the as-synthesized products obtained with various Ca contents (*Y* = 0.25–1.68) in the reactant compacts. As can be seen, in addition to the phase of CASIN (JCPDS No. 39-0747), Ca-α-SiAlON (JCPDS No. 33-0261), AlN (JCPDS No. 89-3446), CaO (JCPDS No. 17-0912), Eu_3_N_2_ (JCPDS No. 89-4095), Ca_2_Al_2_SiO_7_ (JCPDS No. 89-1489) and residual Eu_2_O_3_ (JCPDS No. 43-1009) were also detected. As will be described later, the characteristic emission of AlN:Eu^2+^ was measured at *Y* = 0.25 and that of Ca-α-SiAlON:Eu^2+^ was measured at *Y* = 0.25 and *Y* = 0.75, indicating that AlN:Eu^2+^ and Ca-α-SiAlON:Eu^2+^ were formed at *Y* = 0.25 and *Y* = 0.25–0.75, respectively. (As will be described later, the formation of AlN:Eu^2+^ and Ca-α-SiAlON:Eu^2+^ was also confirmed by TEM-EDS and SAED measurements. In addition, note that the XRD angles of AlN and AlN:Eu^2+^ are very close to each other and thus the two compounds cannot be distinguished on XRD patterns [13,14,15]. The same is the case of Ca-α-SiAlON and Ca-α-SiAlON:Eu^2+^ [16,17,18].) The XRD peak intensity of CASIN is seen to increase with increasing *Y* to a maximum at *Y* = 1.50 but begins to decrease as *Y* is further increased to 1.68, indicating that the formation of CASIN continuously increases as the Ca content is increased from 0.25 to 1.50 but begins to decrease as the Ca content is further increased to 1.68. (The volume percentages of AlN, CaAlSiN_3_ and Ca-α-SiAlON phases formed during synthesis with various Ca contents were estimated based on the XRD measurements and shown in Figure 2.) As mentioned previously, Ca_2_Al_2_SiO_7_, CaO and residual Eu_2_O_3_ can be removed by washing the product with an acid. Figure 1g shows the XRD pattern of the product (synthesized with a Ca content of 1.50) after this washing. (Note that all the XRD patterns shown in Figure 1 were measured under the same experimental conditions.)

Listed in Table 1 and Table 2 are cationic molar ratios of the products (synthesized with various *Y*) after grinding and washing with an acid, which were obtained by ICP-OES and SEM-EDS analysis, respectively. (Note that the values of the cationic molar ratios were obtained by taking the value of Si to be 1.) As can be seen, the Ca and Eu molar ratios in the products both increase with increasing *Y* (except the SEM-EDS analysis of Eu at *Y* = 1.50 and 1.68), indicating that as *Y* increases, the incorporation of Ca and Eu into the products (*i.e*., Ca-α-SiAlON:Eu^2+^ at *Y* = 0.25–0.75 and CaAlSiN_3_:Eu^2+^ at various *Y* for Ca; and AlN:Eu^2+^ at *Y* = 0.25, Ca-α-SiAlON:Eu^2+^ at *Y* = 0.25–0.75 and CaAlSiN_3_:Eu^2+^ at various *Y* for Eu) are both roughly enhanced. Furthermore, the Ca and Eu molar ratios in the synthesized products are seen to be lower than those in the reactant compositions (except the SEM-EDS analysis of Ca at *Y* = 0.25). In fact, this is consistent with the XRD measurement (Figure 1), in which a by-product CaO, and residual Eu_2_O_3_ were detected in all the as-synthesized products.

**Figure 3:**
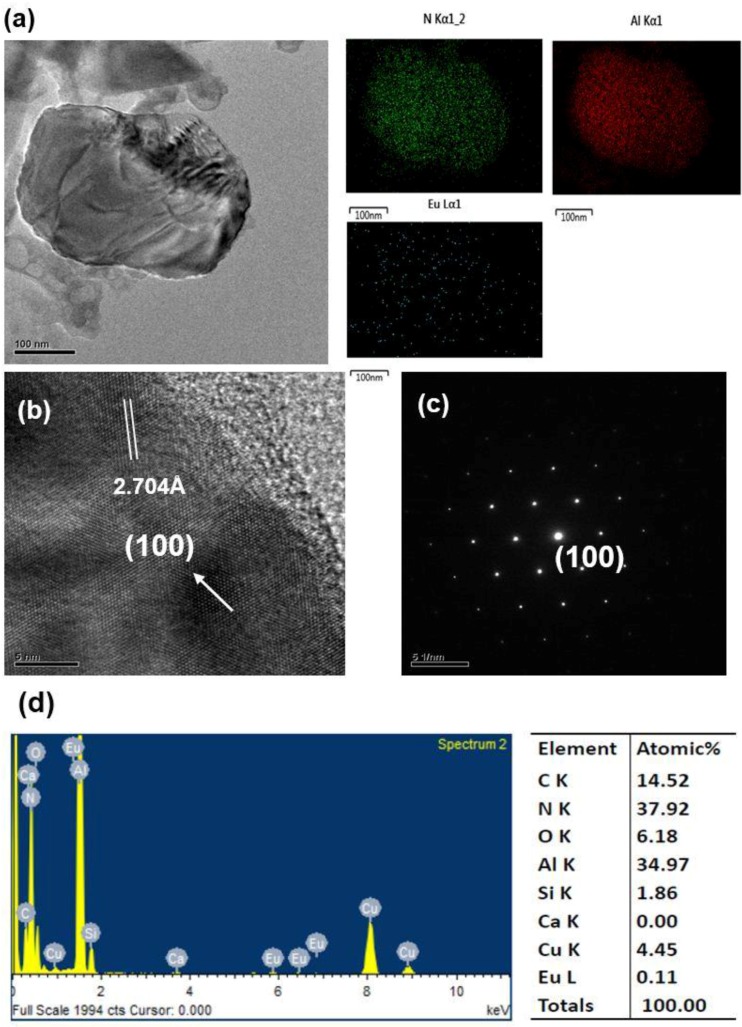
(**a**) TEM image and corresponding element mapping of Al, N, and Eu; (**b**) HRTEM image; (**c**) SAED pattern; and (**d**) element analysis of a plate-like particle (showing it to be AlN:Eu^2+^) in a product synthesized with Ca:Al:Si:Si_3_N_4_:Eu_2_O_3_:NaN_3_:NH_4_Cl = 0.25:1.0:0.25:0.25:0.02:3.5:0.6.

**Figure 4:**
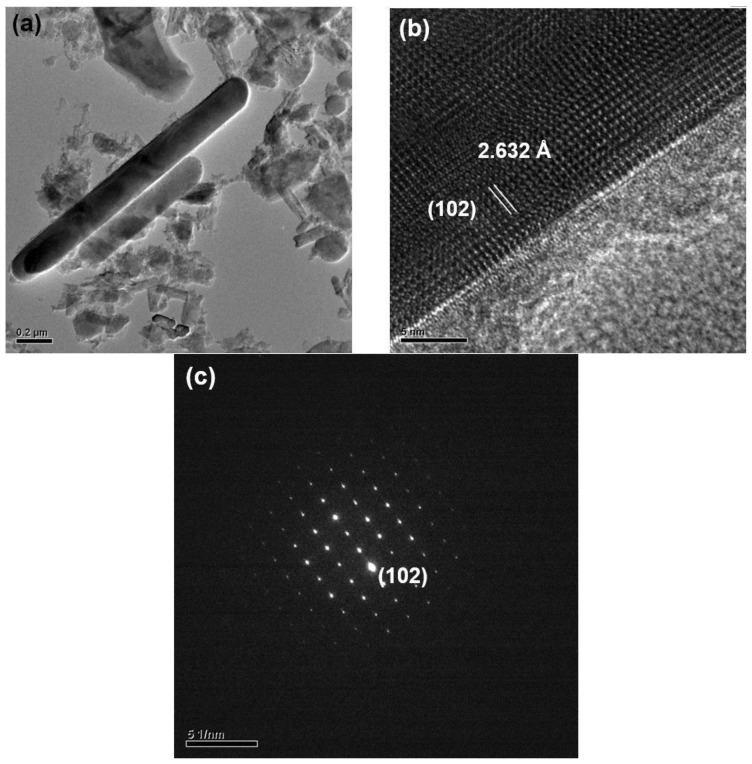
(**a**) TEM image; (**b**) HRTEM image; and (**c**) SAED pattern of a bar-like particle (showing it to be Ca-α-SiAlON:Eu^2+^) in a product synthesized with Ca:Al:Si:Si_3_N_4_:Eu_2_O_3_:NaN_3_:NH_4_Cl = 0.25:1.0:0.25:0.25:0.02:3.5:0.6.

**Figure 5:**
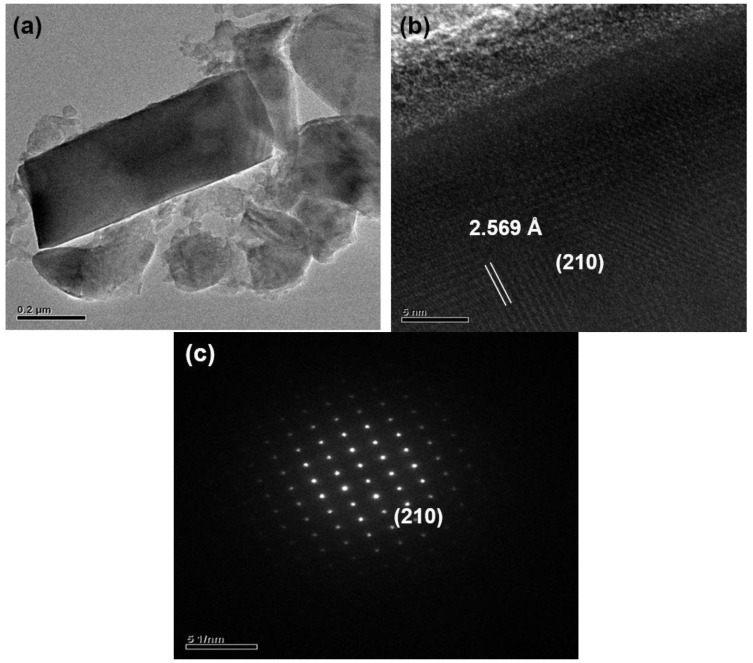
(**a**) TEM image; (**b**) HRTEM image; and (**c**) SAED pattern of a plate-like particle (showing it to be Ca-α-SiAlON:Eu^2+^) in a product synthesized with Ca:Al:Si:Si_3_N_4_:Eu_2_O_3_:NaN_3_:NH_4_Cl = 0.25:1.0:0.25:0.25:0.02:3.5:0.6.

**Figure 6:**
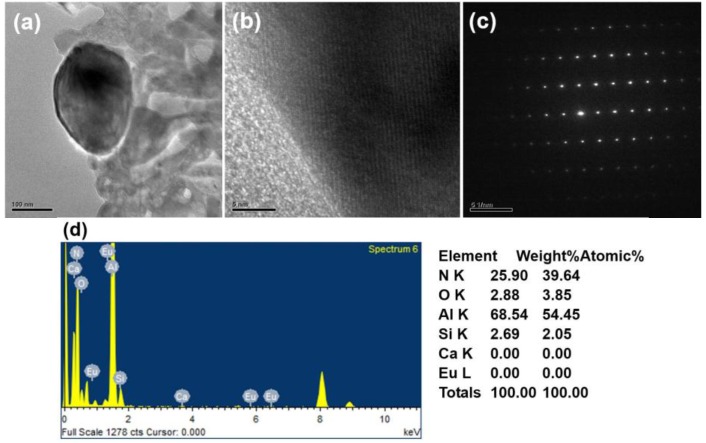
(**a**) TEM image; (**b**) HRTEM image; (**c**) SAED pattern; and (**d**) element analysis of a plate-like particle (showing it to be AlN) in a product synthesized with Ca:Al:Si:Si_3_N_4_:Eu_2_O_3_:NaN_3_:NH_4_Cl = 0.75:1.0:0.25:0.25:0.02:3.5:0.6.

**Figure 7:**
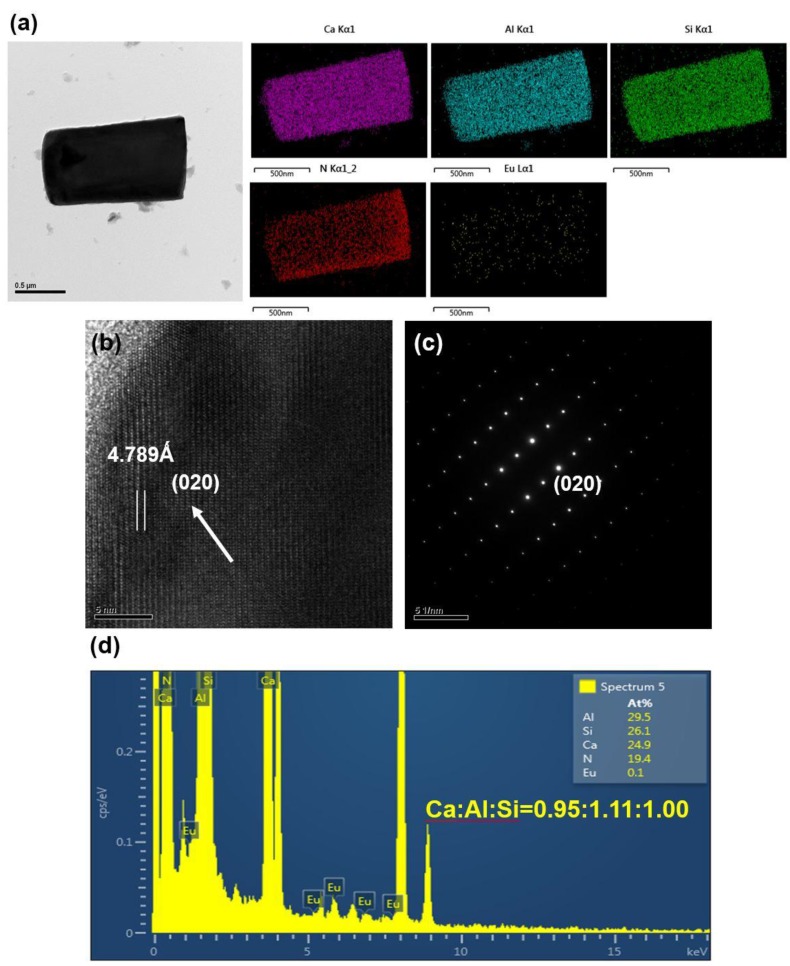
(**a**) TEM image and corresponding element mapping of Ca, Al, Si, N, and Eu; (**b**) HRTEM image; (**c**) SAED pattern; and (**d**) element analysis of a bar-like particle (showing it to be CaAlSiN_3_:Eu^2+^) in a product synthesized with Ca:Al:Si:Si_3_N_4_:Eu_2_O_3_:NaN_3_:NH_4_Cl = 1.5:1.0:0.25:0.25:0.02:3.5:0.6.

**Figure 8:**
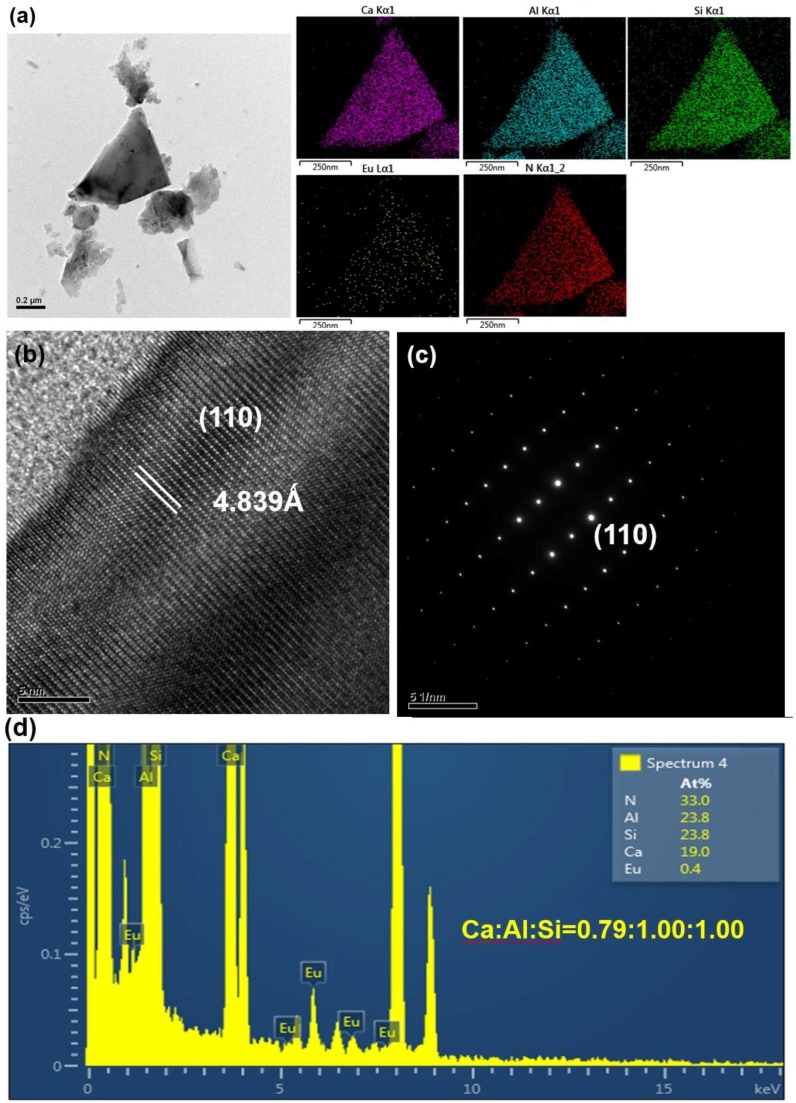
(**a**) TEM image and corresponding element mapping of Ca, Al, Si, N, and Eu; (**b**) HRTEM image; (**c**) SAED pattern; and (**d**) element analysis of a plate-like particle (showing it to be CaAlSiN_3_:Eu^2+^) in a product synthesized with Ca:Al:Si:Si_3_N_4_:Eu_2_O_3_:NaN_3_:NH_4_Cl = 1.5:1.0:0.25:0.25:0.02:3.5:0.6.

**Figure 9:**
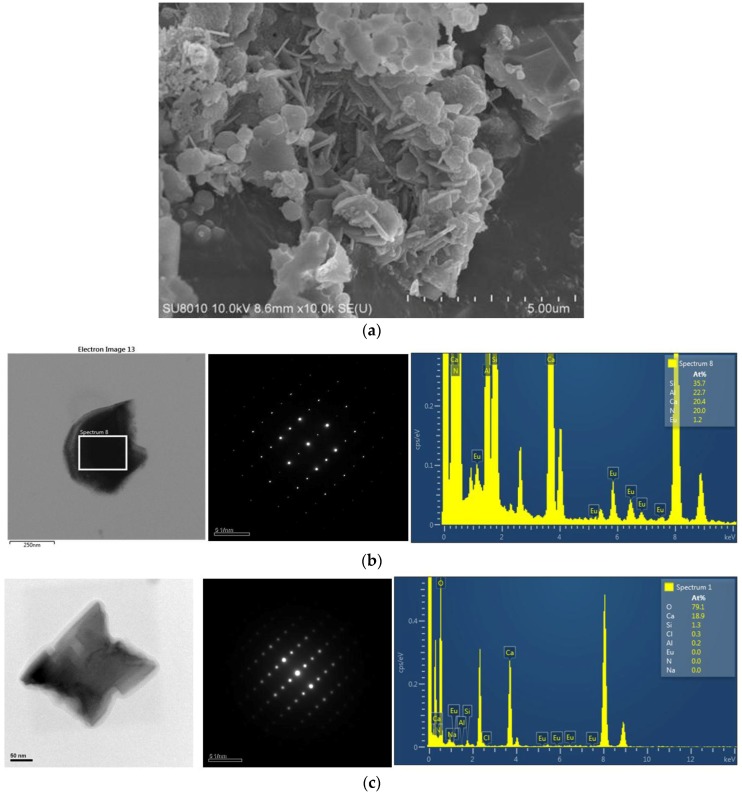
SEM and TEM images, SAED patterns and element analysis of agglomerated fine particles synthesized with Ca:Al:Si:Si_3_N_4_:Eu_2_O_3_:NaN_3_:NH_4_Cl = 1.0:1.0:0.25:0.25:0.02:3.5:0.6. (**a**) SEM image of agglomerated fine particles; (**b**) TEM image, SAED pattern and element analysis of an agglomerated fine particle (showing it to be CaSiAlN_3_:Eu^2+^); (**c**) TEM image, SAED pattern and element analysis of an agglomerated fine particle (showing it to be CaO).

After grinding with a motar and pestle (for 3 min) and washing with an acid, the products were observed to be composed mainly of bar-like, plate-like and agglomerated fine particles. Figure 3, Figure 4, Figure 5, Figure 6, Figure 7, Figure 8 and Figure 9 show the TEM and HRTEM (high resolution transmission electron microscopy) images, SAED patterns and EDS element mapping of the bar-like and plate-like particles in the products synthesized with *Y* = 0.25 (Figure 3, Figure 4 and Figure 5), 0.75 (Figure 6) and 1.50 (Figure 7 and Figure 8). The SAED patterns shown in Figure 4c and Figure 7c indicate that the bar-like crystals can be Ca-α-SiAlON:Eu^2+^ or CaAlSiN_3_:Eu^2+^ and those shown in Figure 3c, Figure 5c, Figure 6c and Figure 8c indicate that the plate-like crystals can be AlN:Eu^2+^, Ca-α-SiAlON:Eu^2+^, AlN or CaAlSiN_3_:Eu^2+^. (Note that the morphologies observed here are in consistency with those reported in other studies [13,14,15,16,17,18,19,20].) The element mapping of each crystal is consistent with the compound revealed by the corresponding SAED pattern. In addition, similar measurements (Figure 9) indicate that the agglomerated fine particles can be CaO, Eu_3_N_2_ or CaAlSiN_3_:Eu^2+^. When *Y* ≤ 1.00, it is found by SEM observation that plate-like and agglomerated fine particles are the major types of particles (with small amounts of bar-like particles). With increasing *Y* in this region (*Y* ≤ 1.00), the amount of plate-like particles decrease while that of agglomerated fine particles increases and at *Y* = 1.00, agglomerated fine particles are the most abundant type of particles. As *Y* is increased from 1.00 to 1.50, the amount of bar-like particles increases while that of agglomerated fine particles greatly decreases. As *Y* is further increased to 1.68, the amount of bar-like particles decreases while that of plate-like particles increases. Among all the samples obtained with various *Y*, the one with *Y* = 1.50 has the most abundant bar-like particles.

### 2.2. Effects of Ca Content on Photoluminescence Properties

**Figure 10:**
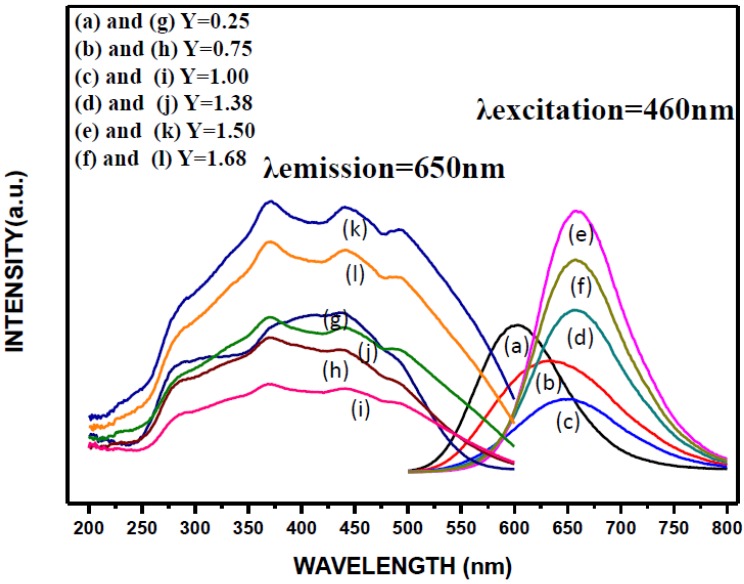
The emission ((**a**); (**b**); (**c**); (**d**); (**e**); and (**f**), excited by λ = 460 nm) and excitation ((**g**); (**h**); (**i**); (**j**); (**k**); and (**l**)) spectra of the phosphors synthesized with various Ca contents.

Figure 10 shows the excitation and emission spectra of the products synthesized with various Ca contents. The excitation spectra were obtained by measuring the emission at 650 nm and the emission spectra were measured by excitation at 460 nm. As can be seen, the wavelength regions of the excitation spectra are all similar (from ~225 to ~600 nm) for various Ca contents. However, their intensity decreases as the Ca content is increased from 0.25 to 1.00 but increases as the Ca content is increased from 1.00 to 1.50 and then decreases again as the Ca content is further increased to 1.68.The excitation spectra all consist of two main absorption bands: The one in the range of 225–400 nm is ascribed to the host lattice excitation due to transition from the valence to the conduction band. The other excitation band being in the range of 400–600 nm is assigned to the 4f^7^→4f^6^5d^1^ transition of the Eu^2+^ ion as a result of crystal field splitting [4,17,18].

**Figure 11:**
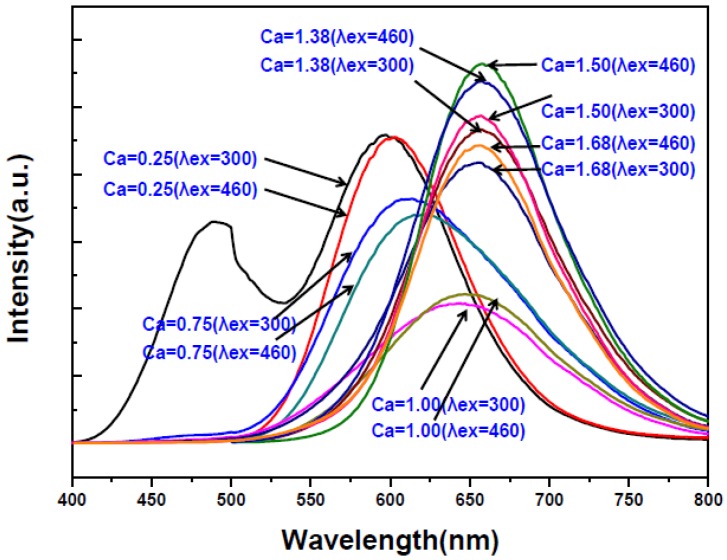
Emission spectra of the products synthesized with various Ca contents under excitation of λ = 300 and 460 nm.

As mentioned previously, the XRD measurements (Figure 1) show that at low Ca contents (*i.e*., *Y* = 0.25 and 0.75), the phases of Ca-α-SiAlON (including Ca-α-SiAlON:Eu^2+^ and Ca-α-SiAlON), AlN (including AlN:Eu^2+^ and AlN), CaO, Eu_3_N_2_, Ca_2_Al_2_SiO_7_ and residual Eu_2_O_3_ were detected in addition to the phase of CaAlSiN_3_ [21]. Among these, CaAlSiN_3_, Ca-α-SiAlON and AlN have been reported to be possible host lattices forming phosphors activated by Eu^2+^ [10,11,12,13,14,15,16,17,18,19,20]. Among these three possible phosphors, AlN:Eu^2+^ cannot be excited by *λ* = 460 nm but can be excited by λ = 300 nm; however, CaAlSiN_3_:Eu^2+^ and Ca-α-SiAlON:Eu^2+^ can both be excited by λ = 300 and 460nm [22,23,24,25,26,27,28,29,30,31,32]. For this reason, the emission spectra of the synthesized products (after grinding and acid washing) by excitation at λ = 300 nm were also measured and the results (together with the emission spectra by excitation at λ = 460 nm as shown in Figure 10) are shown in Figure 11. As can be seen at *Y* = 0.25, the emission spectra excited by λ = 300 nm has two peaks while that by λ = 460 nm has only one peak. The additional peak (centered at 480 nm) is thus believed to be generated by AlN:Eu^2+^. (Note that this emission is in consistency with the observation reported in many other studies [13,14,15].) However, this peak was not measured at *Y* ≥ 0.75 (see Figure 11) perhaps due to the great decrease in the formation of AlN phase at *Y* ≥ 0.75. One other possible reason may be that the doping of AlN with Eu^2+^ (by substitution for Al^3+^) is more difficult than that of Ca-α-SiAlON or CaAlSiN_3_ with Eu^2+^ (by substitution for Ca^2+^). The increasing formation of CaAlSiN_3_ phase at *Y* ≥ 0.75 thus suppresses the formation of AlN:Eu^2+^. (Note the charge difference between Al^3+^ and Eu^2+^ and the differences in radius among the three ions: Al^3+^, 0.50Ǻ; Ca^2+^, 1.00Ǻ and Eu^2+^, 1.17Ǻ.)

**Figure 12:**
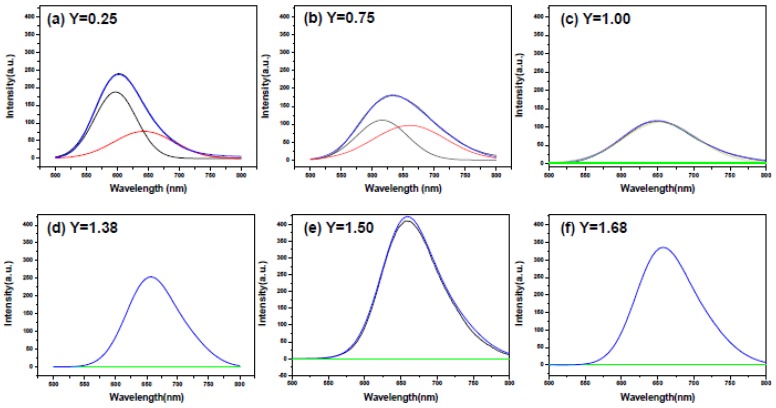
Gaussian deconvolution of photoluminescence emission spectra (by excitation of λ = 460 nm) of the products synthesized with various Ca contents.

Since the emission spectra excited by λ = 460 nm at *Y* ≤ 0.75 appear asymmetric, Gaussian deconvolution was made to all the emission spectra excited by λ = 460 nm and the results are shown in Figure 12. As can be seen, two Gaussian peaks can be deconvoluted for the emission spectra at *Y* = 0.25 and 0.75 (under excitation of λ = 460 nm) by best least-square fit. The high energy emission peaks (centered at ~600 nm) of the deconvoluted emission spectra are believed to be due to Ca-α-SiAlON:Eu^2+^ and the low energy emission peaks (centered at ~650 nm) are due to CaAlSiN_3_:Eu^2+^.

Figure 12 also shows the absence of the high energy emission peak in all the emission spectra with *Y* ≥ 1.0 and all the emission spectra (with *Y* ≥ 1.0) to be single broad band emission [33,34]. The absence of the high energy emission peak is in consistency with the fact that Ca-α-SiAlON phase was not detected by XRD measurement when *Y* ≥ 1.0 (Figure 1) and the single broad band emission (centered at ~650 nm) is apparently generated by CaAlSiN_3_:Eu^2+^. As can be seen in Figure 10, the emission intensity increases as the Ca content is increased from 1.00 to 1.50 but begins to decrease as the Ca content is further increased to 1.68. This may be explained by that the formation of CaAlSiN_3_ phase is increased as *Y* is increased from 1.00 to 1.50 but begins to decrease as *Y* is further increased to 1.68 as revealed by the XRD measurement (Figure 1) described previously. Besides, as described previously, the formation of the bar-like crystals (identified as CaAlSiN_3_:Eu^2+^ at *Y* ≥ 1.00) increases as *Y* is increased from 1.00 to 1.50 but begins to decrease as *Y* is further increased to 1.68. This suggests that the bar-like morphology of CaAlSiN_3_:Eu^2+^ may have stronger emission than the other two morphologies (*i.e*., plate-like and agglomerated fine particles of CaAlSiN_3_:Eu^2+^), thus leading to the above mentioned variation of the emission intensity from *Y* = 1.00 to *Y* = 1.68.

## 3. Experimental Section

As mentioned previously, the present study was carried out by employing the combustion synthesis process developed in our previous study [10] for the synthesis of CASIN. Listed in Table 3 are the characteristics of the reagents used in this study. Calcium, aluminum, silicon, europium oxide, sodium azide (NaN_3_), ammonium chloride and silicon nitride powders were used as the starting materials. To study the effect of Ca content on the formation of CASIN and its photoluminescence property, the molar ratio of Ca (denoted by *Y*) varies from 0.25 to 1.68 while those of others were kept constant as Al:Si:Si_3_N_4_:Eu_2_O_3_:NaN_3_:NH_4_Cl = 1:0.25:0.25:0.02:3.5:0.6. These starting materials were thoroughly mixed in the desired proportions and then pressed into cylindrical compacts (referred to as reactant compacts) with 17 mm in diameter and ~16 mm in length. The reactant compact thus obtained was then wrapped up with an igniting agent *(i.e*., a mixed powder of Mg and Fe_3_O_4_ at 4:1 molar ratio) to obtain a larger cylindrical compact (referred to as a wrapped compact) with 30 mm in diameter and ~30 mm in length (These compacts were prepared following the procedures described in our previous study [10]).

The combustion synthesis reactor used in this study has been described and shown schematically in our previous studies [35,36] and thus is not repeated here. The wrapped compact was placed on a height adjustable stage which was adapted so that the top surface of the compact was about 5 mm below the tungsten heating coil. The reactor was evacuated to 65 Pa by flushing with nitrogen between the evacuations. After the evacuation, the reactor was backfilled with nitrogen to the desired pressures. The combustion reaction was ignited by heating the top surface of the compact for ~10 s by applying an electrical power of ~1 KW to the heating coil.

After combustion, the igniting agent was converted to MgO + Fe, which was loosely attached to the interior product. The interior product could thus be easily separated from the combustion product of the igniting agent. The as-synthesized products contained byproducts, AlN, Ca_2_Al_2_SiO_7_, Eu_3_N_2_, CaO and small amounts of unreacted Eu_2_O_3_. Ca_2_Al_2_SiO_7_, CaO and small amounts of unreacted Eu_2_O_3_ could be removed by washing the products with an acid (e.g., 32 wt. % HCl aqueous solution as used in this study). The crystalline phase of the product was identified by X-ray diffraction (D8 Discover, Bruker Axs Gmbh, Karlsruhe, Germany) using Cu Kα radiation operating at 40 kV and 40 mA. The powder diffraction patterns were obtained using a Bruker Axs Nanostar Universal System coupled with an IuS-type X-ray tube for a microfocus X-ray source with a wavelength of 0.154 nm. The diffraction signals were recorded on an image plate with an exposure period of 30 min. The data was collected in the range of 20° ≤ 2θ ≤ 80°. Scanning electron micrographs (SEM) together with energy-dispersed X-ray spectroscopy (EDS) measurements were performed on a field-emission scanning electron microscope (SU-8100, Hitachi, Japan). The cationic compositions of the synthesized product were analyzed by inductively coupled plasma optical emission spectrometry (ICP-OES, Agilent 725, Santa Clara, CA, USA). The excitation and emission spectra were measured at room temperature using a Fluorescent Spectrophotometer (F-7000, Hitachi, Japan) with a 150 W xenon lamp at a scanning speed of 1200 nm/min. High resolution transmission electron microscopy (*i.e*., HRTEM) images, selected area electron diffraction (SAED) patterns and element mapping were obtained on an transmission electron microscope (JEM-2100F, JEOL, Tokyo, Japan) equipped with an energy-dispersive X-ray spectroscope (EDS) system operating at 200 kV.

## 4. Conclusions

Ca content in the reactant mixture (expressed as molar ratio “*Y*” in Ca:Al:Si:Si_3_N_4_:Eu_2_O_3_:NaN_3_:NH_4_Cl = *Y*:1.0:0.25:0.25:0.02:3.5:0.6) was found to affect significantly the formation and photoluminescence properties of CASIN (CaAlSiN_3_:Eu^2+^) phosphor by combustion synthesis. At *Y* = 0.25, AlN:Eu^2+^ and Ca-α-SiAlON:Eu^2+^ were detected (by XRD) to be formed as major phosphor products. Formation of Ca-α-SiAlON:Eu^2+^ was found to be decreased as *Y* was increased to 0.75 and AlN:Eu^2+^ and Ca-α-SiAlON:Eu^2+^ were not detected at *Y* ≥ 0.75 and ≥1.00, respectively. Formation of CASIN was found to be increased with increasing *Y* as *Y* was increased from 0.25 to 1.50 but began to decrease as *Y* was further increased to 1.68. The emission intensity of CASIN was found to vary with *Y* in a similar trend to its extent of formation. Three major types of morphology were observed for the synthesized products: plate-like crystals, bar-like crystals and agglomerated fine particles. AlN:Eu^2+^ was found to be formed as plate-like crystals, Ca-α-SiAlON:Eu^2+^ was formed as both plate-like and bar-like crystals, and CASIN was formed in all the three types of morphology. Among the three types of morphology of CASIN, the bar-like morphology seems to give a stronger emission than the other two. The Ca and Eu contents (expressed as molar ratios) in the synthesized products were found to increase roughly with increasing *Y* but were both lower than the respective Ca and Eu contents in the reactant mixtures.

## Figures and Tables

**Table 1:** ICP-OES (inductively coupled plasma optical emission spectrometry) analysis of cationic molar ratios of the products (synthesized with various *Y*) after grinding and washing with an acid (also shown are the reactant compositions).

Reactant Composition			Cationic Molar Ratios of the Products				
Ca (*Y*)	Al	Si	Eu	Ca	Al	Si	Eu
0.25	1.00	1.00	0.02	0.12	1.06	1.00	0.010
0.75	1.00	1.00	0.02	0.35	1.19	1.00	0.011
1.00	1.00	1.00	0.02	0.57	1.14	1.00	0.013
1.38	1.00	1.00	0.02	0.64	1.15	1.00	0.014
1.50	1.00	1.00	0.02	0.77	1.18	1.00	0.015
1.68	1.00	1.00	0.02	0.88	1.11	1.00	0.018

**Table 2:** SEM-EDS (scanning electron micrographs (SEM) together with energy-dispersed X-ray spectroscopy (EDS)) analysis of cationic molar ratios of the products (synthesized with various *Y*) after grinding and washing with an acid (also shown are the reactant compositions).

Reactant Composition			Cationic Molar Ratios of the Products				
Ca (Y)	Al	Si	Eu	Ca	Al	Si	Eu
0.25	1.00	1.00	0.02	0.26	0.68	1.00	0.004
0.75	1.00	1.00	0.02	0.39	0.73	1.00	0.006
1.00	1.00	1.00	0.02	0.60	0.71	1.00	0.007
1.38	1.00	1.00	0.02	0.83	0.81	1.00	0.013
1.50	1.00	1.00	0.02	0.86	0.87	1.00	0.013
1.68	1.00	1.00	0.02	0.88	0.90	1.00	0.011

**Table 3:** Characteristics of the Reagents Used in this Study.

Reagent	Particle Size (um)	Purity (%)	Source
Si	1–5	99	Alfa Aesar (Ward Hill, MA, USA)
Ca	<963	99.5	Alfa Aesar (Heysham, England)
Eu_2_O_3_	3.0	99.999	Baogang Group (Baotou, China)
NaN_3_	d_50_ ≈ 150	99	Johnson Matthey (Tokyo, Japan)
NH_4_Cl	-	99	Panreac (Barcelona, Spain)
Al	d_50_ ≈ 3	99	First Chemical Work (Taipei, Taiwan)
α-Si_3_N_4_	d_50_ ≈ 10	99.999	Seminc Company (Ward Hill, MA, USA)
Mg	d_50_ ≈ 20	99	Nihon Shiyaku (Osaka, Japan)
Fe_3_O_4_	102	99	Nihon Shiyaku (Osaka, Japan)
N_2_	Gas	99	Yun Shan (Tainan, Taiwan)
